# Bushenhuoxue formula attenuates cartilage degeneration in an osteoarthritic mouse model through TGF-β/MMP13 signaling

**DOI:** 10.1186/s12967-018-1437-3

**Published:** 2018-03-20

**Authors:** Ping-er Wang, Lei Zhang, Jun Ying, Xing Jin, Cheng Luo, Shibing Xu, Rui Dong, Luwei Xiao, Peijian Tong, Hongting Jin

**Affiliations:** 1 Institute of Orthopaedics and Traumatology, The First Affiliated Hospital of Zhejiang Chinese Medical University, No. 548 Binwen Road, Binjiang District, Hangzhou, 310053 Zhejiang People’s Republic of China; 2 The First College of Clinical Medicine, Zhejiang Chinese Medical University, Hangzhou, 310053 Zhejiang People’s Republic of China; 3 Department of Orthopaedics and Traumatology, Wangjiang Sub-District Community Health Service Center, Hangzhou, 310016 Zhejiang People’s Republic of China; 4 Department of Orthopaedic Surgery, The First Affiliated Hospital of Zhejiang Chinese Medical University, Hangzhou, 310006 Zhejiang People’s Republic of China

**Keywords:** Bushenhuoxue formula, Osteoarthritis, Conditional knockout, Articular cartilage, TGF-β/MMP13 signaling

## Abstract

**Background:**

Articular cartilage degeneration plays a key role in the pathogenesis of osteoarthritis (OA). Bushenhuoxue formula (BSHXF) has been widely used in the treatment of OA in clinics. However, the molecular mechanisms responsible for the chondroprotective effect of BSHXF remain to be elucidated. The purpose of this study was to explore the effects of BSHXF on OA mice model.

**Methods:**

In this study, we investigated the effects of BSHXF on destabilization of the medial meniscus (DMM)-induced chondrocyte degradation in OA mice model. At 12 weeks post-surgery, the joints were harvested for tissue analyses, including histology, histomorphometry, TUNEL, OARSI scoring, micro-CT and immunohistochemistry for COL2, TGFBR2, pSMAD2 and MMP13. Additionally, we also evaluated the effects of BSHXF on *Mmp13* mRNA and protein expression in chondrogenic ATDC5 cells through real-time PCR and Western blot respectively. Moreover, we investigated the chondroprotective effect of BSHXF on mice with *Tgfbr2* conditional knockout (*Tgfbr2*^*Col2ER*^ mice) in chondrocyte, including the relative experiments mentioned above. We transfected *Tgfbr2* siRNA in ATDC5 to further evaluate the changes of *Mmp13* mRNA and protein expression followed by BSHXF treatment.

**Results:**

Amelioration of cartilage degradation and chondrocyte apoptosis were observed in DMM-induced mice, with increases in cartilage area and thickness, proteoglycan matrix, COL2 content and decreases in OARSI score at 12 weeks post surgery. Moreover, the elevated TGFBR2 and pSMAD2, and reduced MMP13 positive cells were also revealed in DMM-induced mice treated with BSHXF. Besides, decreased *Mmp13* mRNA and protein expression were observed inchondrogenic ATDC5 cells culture in serum containing BSHXF. As expected, *Tgfbr2*^*Col2ER*^ mice exhibited significant OA-like phenotype. Interestingly, obvious improvement in articular cartilage structure was still observed in *Tgfbr2*^*Col2ER*^ mice after BSHXF treatment via up-regulated pSMAD2 and down-regulated MMP13 expressional levels in articular cartilage.

**Conclusions:**

BSHXF could inhibit cartilage degradation through TGF-β/MMP13 signaling, and be considered a good option for the treatment of OA.

## Background

Osteoarthritis (OA) which is characterized by abnormal extracellular matrix content (EMC) as well as articular surface erosion, is the most common disabling disease of the joint tissue influenced by interactions among age, genetic and mechanical factors [1]. Knee and hip OA has led to one of the principal causes of global disability [2]. There are a variety of treatment options for OA, but much emphasis in its early stage has been placed on pain relief through medication. Major classes of OA pharmaceuticals include non-steroidal anti-inflammatory drugs (NSAIDs), non-specific cyclooxygenase inhibitors, selective cyclooxygenase-2 (COX-II) inhibitors, opiates and non-opioid oral analgesics [3]. All above painkillers are extensively used in the management of pain associated with OA. Unfortunately, the long-term uses of these drugs may induce prominent side effects. Besides, current OA therapies could not ameliorate all the symptoms and are not considered to be disease modifying approaches [4].

Cartilage degradation is a prominent feature of OA and it may be an important pathological event contributing to the disease pathogenesis. While adult mammal’s articular cartilage appears to show a poor ability of repairing itself throughout life [5, 6]. Both human clinical and animal researches have demonstrated that matrix metalloproteinase 13 (MMP13) plays a key role during articular cartilage degeneration. Clinical study showed that osteoarthritic patients with articular cartilage damage have high MMP13 expression in chondrocyte [7]. Animal studies have also revealed that overexpression of *Mmp13* in transgenic mice’s articular cartilage develop a spontaneous OA-like phenotype [8].

The transforming growth factor beta (TGF-β) signaling which is responsible for regulating the synthesis and degradation of extracellular matrix proteins, for controlling the proliferation and differentiation of chondrocytes and for inhibiting it hypertrophy and maturation [9–11]. Dys-regulation of TGF-β signaling in articular cartilage promotes development of OA [12]. Recently, related study has found that inactivation of TGF-β signaling via deletion of TGF-β receptor type II (*Tgfbr2*) gene in chondrocytes leads to the up-regulated expression *Mmp13* which contributes to the cartilage degradation [13], suggesting that *Mmp13* are critical downstream target gene in the TGF-β signaling during the development of OA. Therefore, MMP13 inhibitor such as ALS 1-0635 [14] would be a potential therapeutic strategy for the OA treatment. However, most of these compounds have failed for toxicity, including skin rash and musculoskeletal side effects characterized by joint stiffness and pain. Currently, no MMP inhibitor has been used in clinics [15].

Traditional Chinese medicine (TCM) acts as the promising alternative medicine as a therapeutic option for OA [16]. Bushenhuoxue formula (BSHXF) orally administered as decoction or granule is a traditional Chinese drug that has been clinically used for many years in the first affiliated hospital of Zhejiang Chinese Medical University. Our previous studies have demonstrated that BSHXF could inhibit cartilage degradation in vivo [17]. Although BSHXF has been proved effective in OA treatment, the precise mechanism and drug action target is still poorly understood.

Thus, we hypothesized that the chondroprotective effect of BSHXF based on inhibition of MMP13 through TGF-β/MMP13 signaling in chondrocyte. In this study, we firstly sought to determine the effect of high-performance liquid chromatography (HPLC) standardized BSHXF. Furthermore, we determined the impact of BSHXF in osteoarthritic cartilage in mouse model. We also used *Tgfbr2* conditional knockout (cKO) mice in articular cartilage and determined the changes of articular cartilage structure followed by BSHXF treatment.

## Methods

### Preparation of BSHXF

Herb pieces of BSHXF were offered from pharmacy department of the first affiliated hospital of Zhejiang Chinese Medical University (Hangzhou, China). The extraction process includes two parts: aqueous and ethanol extracts. *Carthamus tinctorius* (L.), *Rehmannia glutinosa* (Libosch.), *Eucommia ulmoides* (Oliv.), *Aconitum carmichaelii* (Debx.), *Lycium barbarum* (L.), *Cornus officinalis* (Sieb.), *Dioscorea opposite* (Thunb.) and *Glycyrrhiza uralensis* (Fisch.) were mixed as a proportion of 1:3:2:2:2:1:2:1 (w/w) and the total dry weight was 42 kg for aqueous extracts. The mixture of herbs above was soaked in 12 volumes of distilled water for 1 h, extracted for 3 times by the reflux method and 1.5 h each time. *Prunus persica* (Batsch.) and *Cinnamomum cassia* (Presl.) were mixed in the ratio of 2:1 (w/w) and the total dry weight was 9 kg for ethanol extracts. These pieces were immersed in 10 volumes of 60% ethanol for 1 h, extracted for 3 times and 1.5 h each time. Then, two types of extract were thoroughly mixed to obtain 15 L medicated solution. The solution was concentrated to 2 g crude drug/mL for further usage according to the daily dose and kept in − 20 °C to preserve.

### Compound identification of BSHXF

**Fig. 1:**
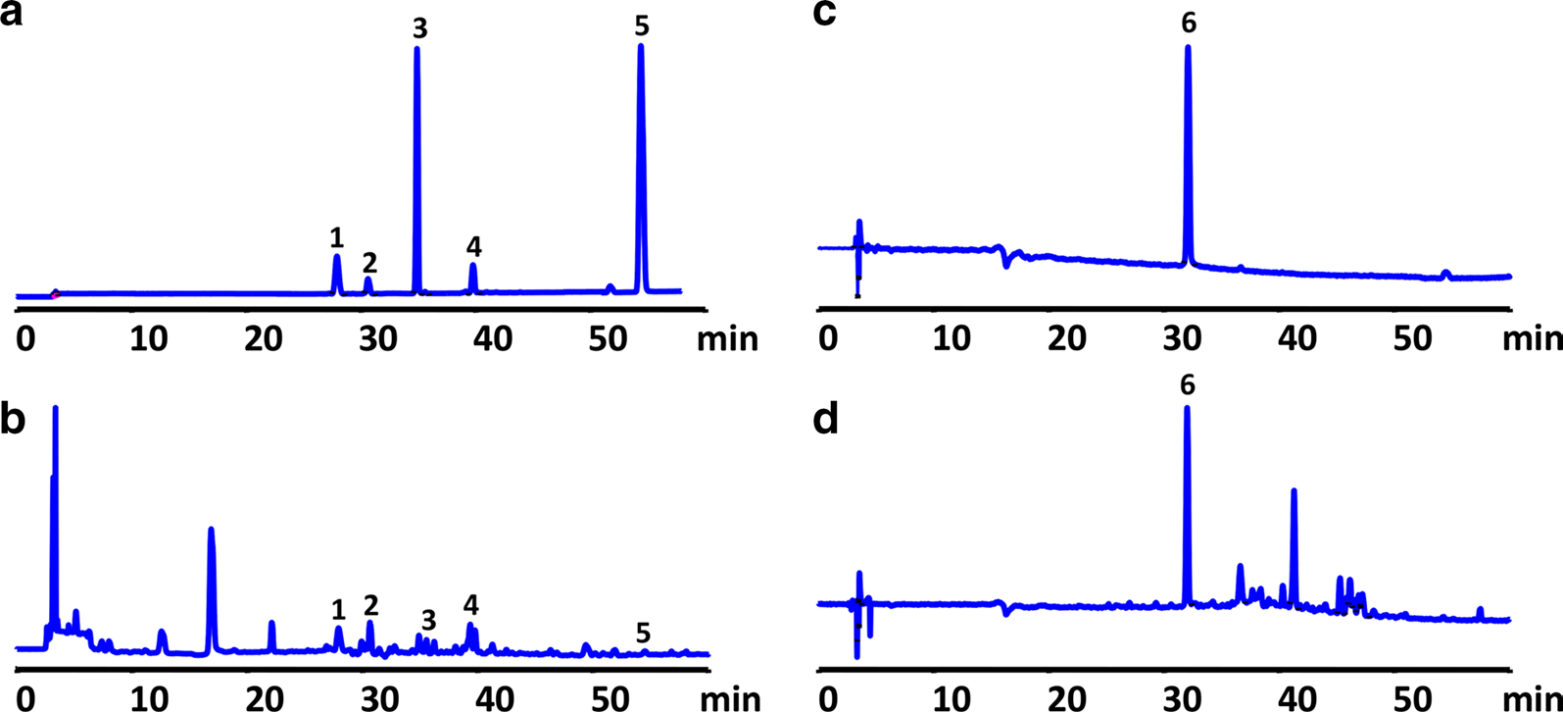
HPLC chromatographic fingerprint analysis of BSHXF sample. **a** Mixed reference standard substance. **b** Peaks 1–5: loganin (2.42 mg/g), amygdalin (4.07 mg/g), pinoresinol diglucoside (0.76 mg/g), liquiritin (0.79 mg/g), cinnamaldehyde (0.16 mg/g). **c** Reference standard substance. **d** Peak 6: hydroxysafflor yellow A (0.32 mg/g)

Chemical constituents of BSHXF were identified by high performance liquid chromatography (HPLC) which was also used to control the quality. The sample was prepared according to following steps: 20% methanol was added into 1 g BSHXF powder prior to ultrasonic irradiation for 30 min. The mixed solution was then cooled down to room temperature and filter through 0.45 μm microporous membrane. The chromatographic separation was obtained on an Ultimate XB C18 (4.61 × 100 mm, 5 μm) column of which temperature was 25 °C in an Agilent 1200 HPLC system (Agilent Technologies, Sanata, CA, USA). The mobile phase consisted of A: acetonitrile and B: 0.1% phosphoric acid (v/v) at a flow rate of 1.0 mL/min by gradient elution (0–10 min, 1–2% A; 10–13 min, 2–5% A; 13–28 min, 5–15% A; 28–48 min, 15–35% A; 48–60 min, 35–60% A), the sample size of process above was 5 μL. The results were compared at different wavelengths and the HPLC fingerprint was established based on the wavelength detection included a switched multi-wavelength (0–3.5 min, 210 nm; 3.5–6 min, 237 nm; 6–15 min, 210 nm; 15–18 min, 290 nm; 18–29 min, 237 nm; 29–65 min, 210 nm) and a single wavelength (403 nm). As showed in Fig. 1, six chromatogram peaks were identified. From No. 1 to 6, the peaks represent loganin, amygdalin, pinoresinol diglucoside, liquiritin, cinnamaldehyde, hydroxysafflor yellow A, respectively.

### Animals

Both C57BL/6J mice for modeling and Sprague Dawley (SD) rats for serum acquisition were obtained from Shanghai Laboratory Animal Center of Chinese Academy of Science (Grade SPF II, SCXK 2012-0002). *Tgfbr2* gene was specifically knocked out in chondrocyte of mice (C57BL/6J background) based on a Cre/loxP conditional knockout (cKO) allele of the *Tgfbr2* gene. *Col2*-*CreER* mice were crossed with *Tgfbr2*^*fx/fx*^mice to generate *Col2*-*CreER*; *Tgfbr2*^*fx/fx*^ mice (*Tgfbr2*^*Col2ER*^ mice) (Table 1) and all original mice were donated from Rush University (Chicago, IL, USA). Genotyping was performed by polymerase chain reaction (PCR) using a DNA extraction kit (Sigma, St. Louis, MO, USA) tail biopsy tissues with a Eppendorf 5333 PCR System (Eppendorf AG, Hamburg, Germany). PCR primer sequences for genotyping were as follows: primer sequences of *Cre*, forward primer 5′-ATTGCTGTCACTTGGTCGTGGC-3′ and reverse primer 5′-GAAAATGCTTCTGTCCGTTTGC-3′ (200-base-pair PCR product); primer sequences of *Tgfbr2* loxP, forward primer 5′-TAAACAAGGTCCGGAGCCCA-3′ and reverse primer 5′-ACTTCTGCAAGAGGTCCCCT-3′ (Wild-type, 420-base-pair PCR product; homozygotic type, 540-base-pair PCR product). Cre-negative littermates were used as controls. After identification of genotypes, *Tgfbr2*^*Col2ER*^ mice were induced by intraperitoneal injection (i.p.) with tamoxifen (1 mg/10 g body weight/day, for 5 days) (Sigma, St. Louis, MO, USA) at age of 2 weeks [13]. All mice were housed at 21 ± 2 °C with a relative humidity of 40 ± 5%. The room was maintained on a 12 h light/dark cycle with free access to water and lab chow. All studies were approved by the Committee on the Ethics of Animal Experiments of Zhejiang Chinese Medical University.

**Table 1:** Breeding of *Col2*-*CreER*; *Tgfbr2*^*fx/fx*^ mice

Breeding	Desired progeny
(a) *Col2*-*CreER* × *Tgfbr2*^*fx/fx*^	(a) *Col2*-*CreER*; *Tgfbr2*^*fx/wt*^
(b) *Col2*-*CreER*; *Tgfbr2*^*fx/wt *^× *Tgfbr2*^*fx/fx*^	(b) *Col2*-*CreER*; *Tgfbr2*^*fx/fx*^
(c) *Col2*-*CreER*; *Tgfbr2*^*fx/fx *^× *Tgfbr2*^*fx/fx*^	(c) *Col2*-*CreER*; *Tgfbr2*^*fx/fx*^ and *Tgfbr2*^*fx/fx*^

### Surgical preparation

Knee osteoarthritic model was established when the WT mice were 10-week-old by destabilization of the medial meniscus (DMM) and challenge as described previously [18]. Briefly, DMM surgery was performed on the right hind limb of WT mice as follows: (1) after a 3 mm longitudinal incision was made on the medial part of the knee under anesthesia, blunt dissection of the knee extensor muscles and patellar ligament was performed to expose the medial meniscotibial ligament (MMTL); (2) The MMTL was transected to give destabilization of the medial meniscus (DMM); (3) The medial joint capsule was sutured, and the skin was closed. The sham surgery was also performed by a similar surgical approach without manipulating the joint tissue in WT mice.

### Drug administration and experimental groups

All mice were randomly divided into six groups: the sham group, the model group, the BSHXF group, the Cre-negative group, the *Tgfbr2*^*Col2ER*^ group and the *Tgfbr2*^*Col2ER*^ + BSHXF group (n = 10 in each group). BSHXF was orally administered to both WT and *Tgfbr2*^*Col2ER*^ mice once a day after DMM surgery and tamoxifen inducement respectively for 12 consecutive weeks with dose of 0.2 mL/10 g body weight. The dose administered was determined according to the following formula: $D_{\text{M}} \left( {\text{dose per kg body weight}} \right)\, = \,D_{\text{H}} \, \times \,R\, \times \,\left( {{{W_{\text{H}} } \mathord{\left/ {\vphantom {{W_{\text{H}} } {W_{\text{M}} }}} \right. \kern-0pt} {W_{\text{M}} }}} \right),$ as detailed in The methodology of pharmacological experiment. *D*_M_ and *D*_H_ are doses for mice and humans, and *W*_M_ and *W*_H_ are body weights of mice and humans, respectively. *R* is the coefficient of 0.0026 for human mouse equivalent dosage conversion [19]. The mice in sham and Cre-negative group were fed with an equal dosage of 0.9% normal saline. Knee joints samples were harvested after drug intervention for the follow-up experiments.

### Histological analysis

Knee joints harvested for histological analysis were successively fixed in 4% paraformaldehyde for 3 days, decalcified with 14% EDTA solution for 14 days and embedded in paraffin. Three micrometre thick sections were cut longitudinally from the medial compartment of the joints. The sections were stained with Alcian Blue Hematoxylin/Orange G and Toluidine Blue for analysis of gross structural changes. Histomorphometric analysis was performed using OsteoMeasure software (OsteoMetrics, Inc., Atlanta, GA, USA). Cartilage structure degeneration was scored by two blinded observers according to Osteoarthritis Research Society International (OARSI) recommendations [20].

### Micro-CT evaluation

The representative knee joint images were obtained from micro-CT equipment (Skyscan 1176, Bruker microCT N.V., Kontich, Belgium). The pictures were taken with a resolution of 4000 × 2672 pixels and an isotropic voxel size of 9 μm. In addition to the visual assessment of structural pictures, quantitative morphometry indexes were determined from micro-tomographic data based on the 3-D morphometry [21]. The following indexes were determined: (i) bone volume fraction (BV/TV, %), (ii) average trabecular thickness (Tb. Th, mm) and (iii) average trabecular separation (Tb. Sp, mm).

### Immunohistochemistry (IHC) analysis

Three micrometre deparaffinized sections were treated with 0.3% hydrogen peroxide to reduce endogenous peroxidase activity. Then the sections were incubated for 20 min at 95 °C with 0.1 mol/L citrate buffer as antigen retrieval. Non-specific staining was blocked by incubation of normal goat serum (diluted 1:20) (Invitrogen, MD, USA) for 20 min at room temperature. Subsequently, the sections were treated with collagen II (COL2) (diluted 1:1000), matrix metallopeptidase 13 (MMP13) (diluted 1:100), transforming growth factor beta receptor II (TGFBR2) (diluted 1:100) or phosphorylated protein mothers against decapentaplegic homolog 2 (pSMAD2) (diluted 1:100) primary antibodies (Abcam, MA, USA) and further incubated overnight at 4 °C. Secondary biotinylated goat anti-mouse antibody (diluted 1:1000) (Invitrogen, MD, USA) was added for 30 min on the second day. Positive staining of sections was detected by diaminobenzidine solution (Invitrogen, MD, USA) followed by counterstaining with hematoxylin. The primary antibody was not added to negative controls.

The nuclei were considered positive for TGFBR2, pSMAD2 and MMP13 labeling if their immunostains were equal or larger than 50% of the nuclear area. Weak brown stains were excluded from the counting. The rate of TGFBR2, pSMAD2 and MMP13 positive cells were determined using Image-Pro Plus 6.0 (Media Cybernetics, Silver Spring, USA).

### Terminal deoxynucleotidyl transferase dUTP nick end labeling (TUNEL) analysis

TUNEL staining was performed using a staining kit to detect chondrocyte apoptosis. The protocol of TUNEL staining was based on the manufacturer’s instruction (Roche, IN, USA).

### Preparation of serum containing BSHXF

Fifty eight-week-old female SD rats weighted 200 ± 20 g were randomly divided into two equal groups (n = 25 in each group). The rats in BSHXF group were orally administrated with BSHXF (1 mg/10 g body weight/day [19], for 7 days), and normal control group were orally administrated with 0.9% saline at the same dose. Then the blood was collected separately and the serum was reserved via centrifugation at 3000 rpm/min for 10 min. After filtered and inactivated at 56 °C for 30 min, the serum of two groups was stored at − 80 °C for subsequent use.

### Cell culture and treatment

Chondrogenic ATDC5 cells (Riken Cell Bank, Ibaraki, Japan) were cultured in Dulbecco’s modified Eagle’s medium/Ham’s F-12 medium (DMEM/F12; 1:1 mixture) (Life Technologies, MD, USA) containing 10% (v/v) foetal bovine serum (FBS) (Sigma Chemicals, MO, USA), and 1% penicillin and streptomycin (Hyclone, Beijing, China) at 37 °C in a humidified 5% carbon dioxide atmosphere. Confluent ATDC5 cells were stimulated to undergo chondrogenesis by addition of ITS (insulin, transferrin, and selenous acid) (Gibco BRL, MD, USA). Chondrogenesis was induced in ATDC5 cells for 2 weeks. At 80% confluence, chondrogenic ATDC5 cells were exposed to minimal supplement with 7.5% FBS + 2.5% rat serum or serum containing BSHXF. The culture medium was changed every 2–3 days.

### Small interfering RNA (siRNA) transfection

*Tgfbr2*-siRNA and non-silencing siRNA were purchased from Ribo (RiboBio, Guangzhou, China). Active siRNA against *Tgfbr2* used in the in vitro studies had sequences 5′-CCUGUUGCCUGUGUGACUU-3′ (sense) and 3′-GGACAACGGACACACUGAA-5′ (antisense). *Tgfbr2*-siRNA transfection of siRNA was performed using lipofectamine transfection regent 2000 (Invitrogen, Carlsbad, CA), according to the manufacturer’s protocol. Control cells were mock transfected with lipofectamine transfection regent 2000 only. Chondrogenic ATDC5 cells were seeded in a 6-well plate until cells reached 50% confluence. The full culture medium was changed with serum-free and antibiotic-free medium at 20 min before transfection. The cells were incubated with transfection mixtures containing *Tgfbr2*-siRNA or non-silencing siRNA for 6 h, and then the mixtures were replaced with complete culture medium.

### Real-time polymerase chain reaction (PCR) analysis

Total RNA was extracted from differentiated chondrogensis respectively cultured in control serum and serum containing BSHXF using TRIzol (Invitrogen, CA, USA), and cDNA was generated from 2 μg RNA using RevertAid First Strand cDNA Synthesis Kit (Invitrogen, CA, USA) following the manufacturer’s instruction. Real-time PCR was performed for target gene using the SYBR Premix Ex Taq™ II (Takara, Dalian, China) with a QuantStudio™ 7 Flex Real-Time PCR System (Thermo Scientific, MA, USA) according to the manufacturer’s instruction. The specific primers are as followed: primer sequences of *Mmp13*, forward primer 5′-TTTGAGAACACGGGGAAGA-3′ and reverse primer 5′-ACTTTGTTGCCAATTCCAGG-3′; primer sequences of *Actb*, forward primer 5′-GGAGATTACTGCCCTGGCTCCTA-3′ and reverse primer 5′-GACTCATCGTACTCCTGCTTGCTG-3′. Relative *Mmp13* gene expression values were analysed using the comparative 2^−∆∆Ct^ method for relative quantification, which is implemented in the QuantStudio™ 6 and 7 Flex Software (Thermo Scientific, MA, USA). *Actb* was used as an endogenous control gene for PCR normalization concerning the amount of RNA added to the reverse transcription reactions.

### Western blot analysis

Total proteins were collected immediately in lysis buffer containing protease and phosphatase inhibitors, incubated on ice for 30 min and separated on a 12% SDS-PAGE gel. The samples were then transferred onto polyvinylidene fluoride membranes and blocked in 5% milk for 1 h. Immunoblotting was subsequently performed by incubating the membranes overnight at 4 °C with rabbit primary antibodies targeting MMP13 (diluted 1:1000; Abcam, MA, USA). The membranes were incubated with goat anti-rabbit horseradish peroxidase-conjugated secondary antibody (diluted 1:5000; Abcam, MA, USA) at room temperature. The densities of the bands were determined using the Tocan 190 protein assay system (Bio-Rad, CA, USA) and normalized to *Actb*.

### Date analysis

All the data were expressed as mean ± standard deviation. One-way analysis of variance (ANOVA) test was used to compare the means among groups. And the multiple comparisons were performed with least significant difference (LSD) test. A *P* value of less than 0.05 was considered to be statistically significant. The statistical analysis was performed using SPSS 18.0 software (SPSS Inc., Chicago, IL, USA).

## Results

### BSHXF decelerates DMM-induced OA progression

**Fig. 2:**
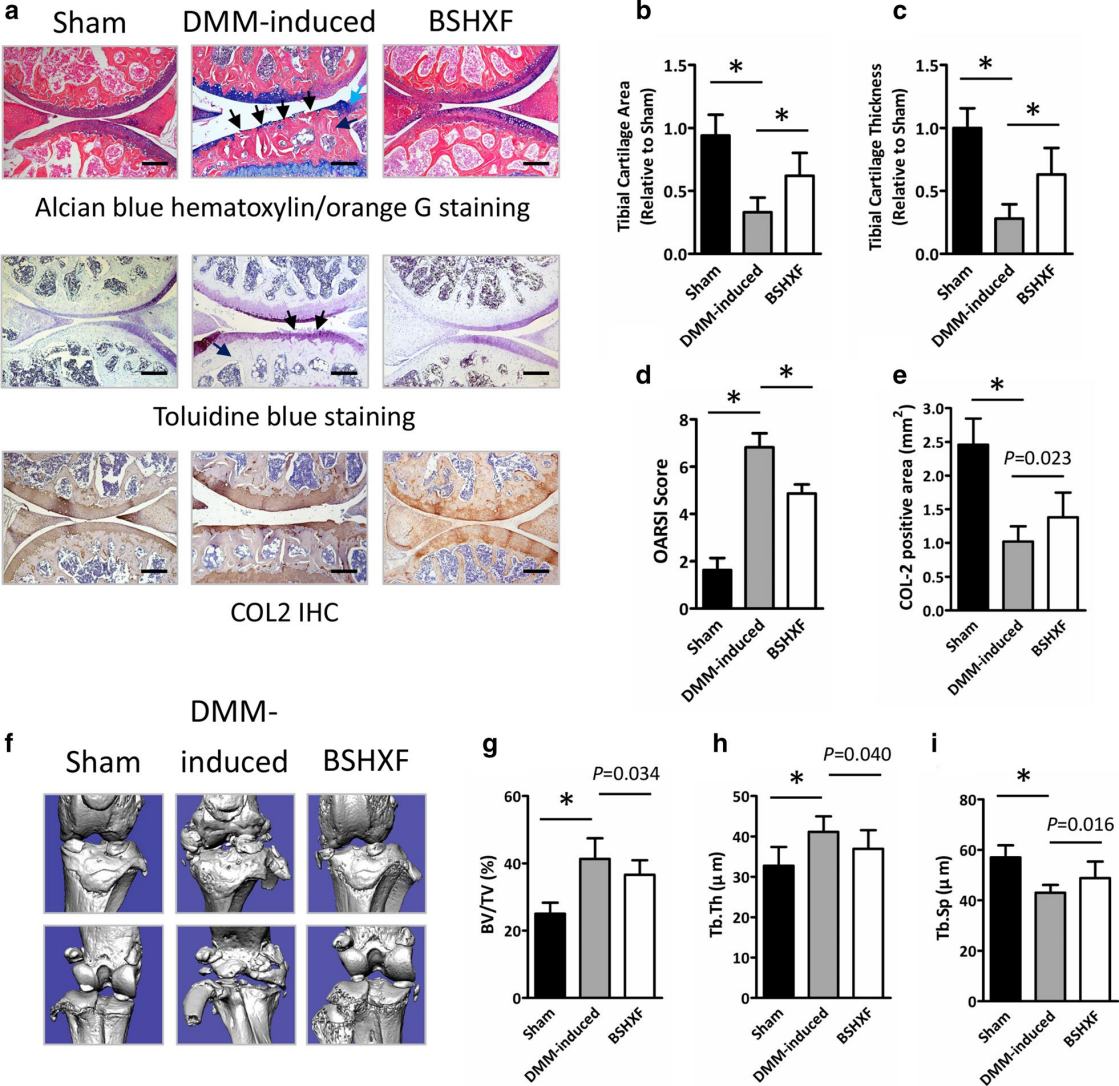
BSHXF protects against cartilage degeneration in DMM-induced mice. **a** Histological knee joint sections (100×) stained using Alcian Blue Hematoxylin/Orange G and Toluidine Blue, and COL2 IHC. Joint degradation are labeled (black arrows: cartilage degradation, blue arrows: osteophyte formation, purple arrows: subchondral sclerosis). Cartilage architecture was evaluated using the Osteomeasure System to determine **b** the tibial cartilage area, **c** tibial cartilage thickness, **d** OARSI scoring and **e** COL2 positive area. **f** Representative micro-CT images. Quantification of the **g** BV/TV, **h** Tb. Th and **i** Tb. Sp by static histomorphometry. Bars represent mean ± SD (n = 10). *P < 0.01. Scale bars = 200 μm

Alcian Blue Hematoxylin/Orange G and Toluidine Blue staining revealed that prominent focal cartilage defects, early osteophyte formation and increases in subchondral bone mass was observed in DMM-induced mice compared to sham group. Additionally, the results of IHC showed lower COL2 content in DMM-induced mice. However, BSHXF dramatically inhibited the cartilage degeneration, COL2 content loss and osteophyte formation (Fig. 2a, e). Histomorphometric analysis showed a significant increased cartilage area (Fig. 2b) and thickness (Fig. 2c) in mice treated with BSHXF (both, *P *< 0.01). We also evaluated the cartilage degradation through OARSI grading, and we found that mice in BSHXF group had significantly lower scores compared to model mice (*P *< 0.01), with significantly reduced OARSI scores after treatment with BSHXF (*P *< 0.01) (Fig. 2d). Consistent with the histological analysis, the result of COL2 IHC also showed an increased COL2 content in DMM-induced mice treated with BSHXF (*P *= 0.023) (Fig. 2a, e). Moreover, the area of trabecular bone in the proximal metaphysis of tibia was evaluated by 3D data analysis software (CTAn, Bruker micro-CT^®^). As shown in Fig. 2f, BSHXF prominently reduced DMM-induced osteophyte formation. Compared to sham mice, up-regulated BV/TV, Tb. Th and down-regulated Tb. Sp were exhibited in model group (all, *P *< 0.01). After BSHXF treatment, the BV/TV and Tb. Th measurements were significantly lower than those of DMM-induced mice (*P *= 0.034, *P *= 0.040) (Fig. 2g, h). Conversely, the Tb. Sp measurements of the BSHXF group were significantly higher than those of DMM-induced mice (*P *= 0.016) (Fig. 2i), suggesting that BSHXF has a positive effect on bone health in DMM-induced mice.

### DMM-induced up-regulation of MMP13 is reduced by BSHXF

**Fig. 3:**
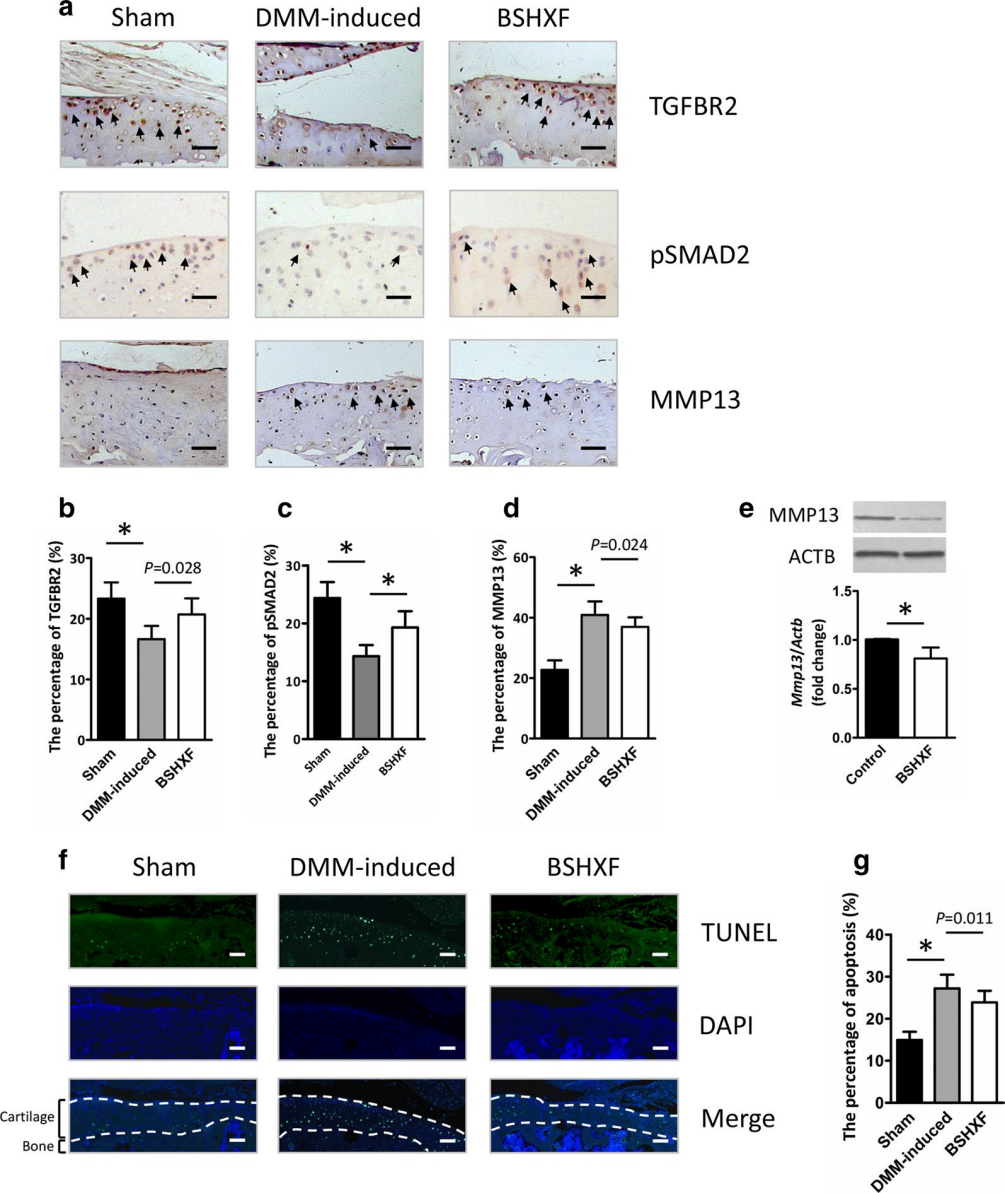
BSHXF reduces MMP13 and elevate TGFBR2 and pSMAD2 expressional levels in osteoarthritic chondrocyte. **a** Knee joint representative IHC image (200×) of TGFBR2, pSMAD2 and MMP13 stained chondrocytes (brown; black arrows) with cell nuclei counterstained with hematoxylin (blue). Quantification of **b** TGFBR2, **c** pSMAD2 and **d** MMP13 as positive cells rate. **e** *Mmp13* mRNA and protein expression inchondrogenic ATDC5 cells. **f** Terminal deoxynucleotidyl transferase dUTP nick end labeling chondrocyte apoptosis (200×). **g** Quantification of TUNEL staining positive cells was performed to evaluate chondrocyte apoptosis. Bars represent mean ± SD (n = 10). *P < 0.01. Scale bars = 100 μm

A key link in articular cartilage erosion and chondrocytes apoptosis is the imbalance of synthesis and degeneration of the extracellular matrix (ECM), an event marked by the expression of proteolytic enzymes such as MMP13, and the important molecular of TGF-β/MMP13 signaling, TGFBR2 and pSMAD2. Accordingly, it is possible that the chondroprotective action of BSHXF documented in Fig. 3 is associated with TGF-β/MMP13 signaling. To address this question, IHC was performed to evaluate TGFBR2, pSMAD2 and MMP13 protein expressional levels in the articular cartilage. As expected, DMM-induced mice had reduced protein expressional level of TGFBR2 and pSMAD2, and elevated levels of MMP13 in cartilage compared to sham mice (both, *P *< 0.01) (Fig. 3a–d). Mice treated with BSHXF exhibited decreased MMP13 positive cells (*P *= 0.024) (Fig. 3d), suggesting that maintaince of cartilage architecture in BSHXF group could be due to inhibition of matrix degradation. Remarkably, elevated TGFBR2 and pSMAD2 positive cells were also observed in BSHXF group (*P *= 0.028, *P *< 0.01) (Fig. 3b, c). The mechanism of BSHXF regulation of *Mmp13* expression was investigated through an in vitro experiment. Consistent with the IHC analysis, we found reduction of *Mmp13* mRNA and protein levels in chondrogenic ATDC5 cells cultured with serum containing BSHXF through real-time PCR and Western blot analysis respectively (Fig. 3e). To investigate chondrocytes apoptosis, we performed TUNEL staining on DMM-induced mice. DMM-induction of broad chondrocyte apoptosis was reduced followed by BSHXF (*P *= 0.011) (Fig. 3f, g). These results indicated that BSHXF may have chondroprotective effects on DMM-induced mice in part due to reduced MMP13 production by chondrocytes residing in cartilage through TGF-β/MMP13 signaling.

### BSHXF prevents OA-like phenotype observed in *Tgfbr2*^*Col2ER*^ mice

**Fig. 4:**
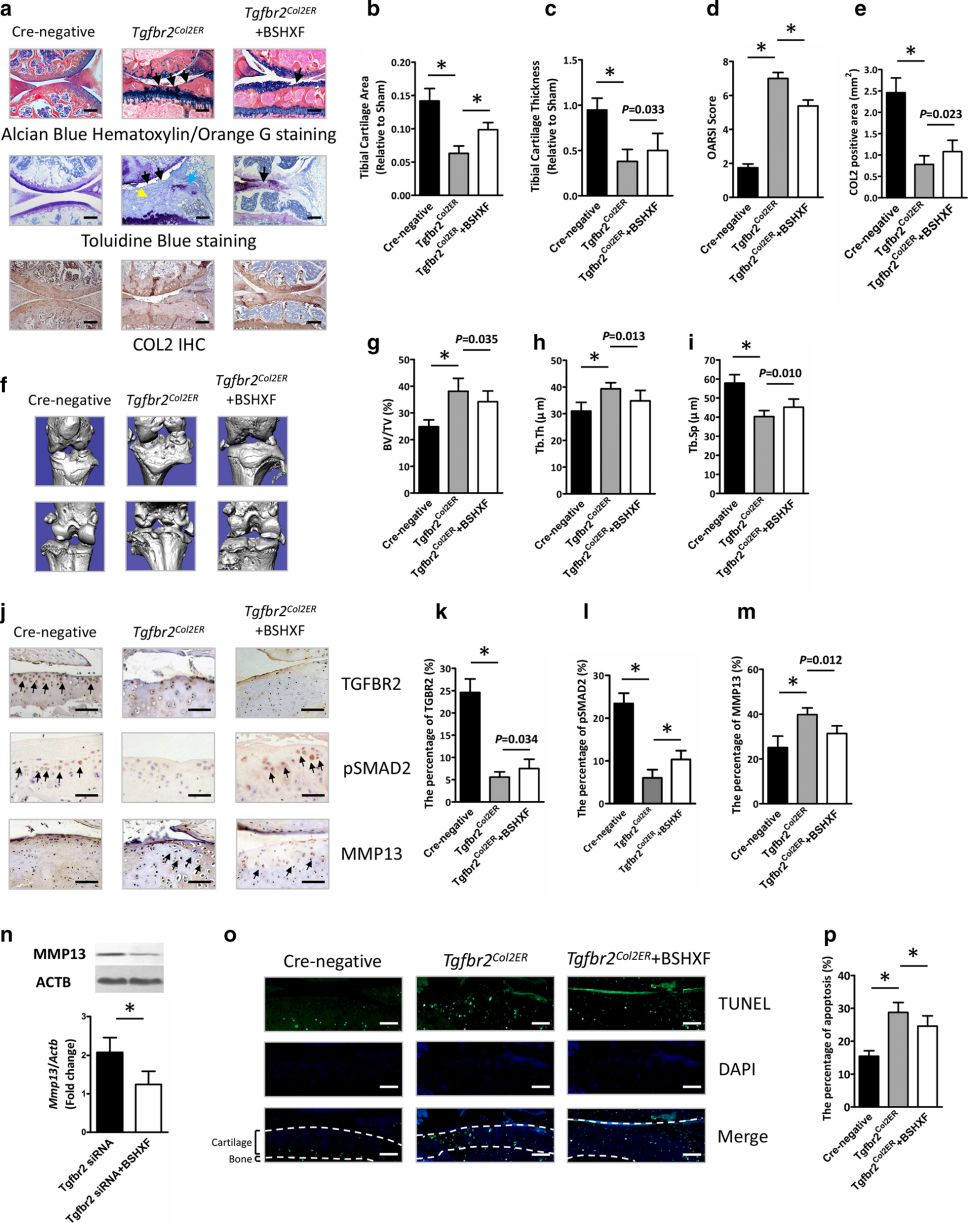
BSHXF decelerates cartilage degeneration in *Tgfbr2*^*Col2ER*^ mice via down-regulations of MMP13. **a** Histological knee joint sections (100×) stained using Alcian Blue Hematoxylin/Orange G and Toluidine Blue, and COL2 IHC. Joint degradation are labeled (black arrows: cartilage degradation, blue arrows: osteophyte formation, purple arrows: subchondral sclerosis). Cartilage architecture was evaluated using the Osteomeasure System to determine **b** the tibial cartilage area, **c** tibial cartilage thickness, **d** OARSI scoring and **e** COL2 positive area. **f** Representative micro-CT images. Quantification of the **g** BV/TV, **h** Tb. Th and **i** Tb. Sp by static histomorphometry. **j** Knee joint representative IHC image (200×) of TGFBR2, pSMAD2 and MMP13 stained chondrocytes (brown; black arrows) with cell nuclei counterstained with hematoxylin (blue). Quantification of **k** TGFBR2, **l** pSMAD2 and **m** MMP13 as positive cells rate. **n** *Mmp13* mRNA and protein expression inchondrogenic ATDC5 cells. **o** Terminal deoxynucleotidyl transferase dUTP nick end labeling chondrocyte apoptosis (200×). **p** Quantification of TUNEL staining positive cells was performed to evaluate chondrocyte apoptosis. Bars represent mean ± SD (n = 10). *P < 0.01. Scale bars = 200 μm

The marked preservation of ECM produced by chondrocytes post-DMM in BSHXF treated mice needed for subsequent investigation of the impact of BSHXF on OA progression in *Tgfbr2*^*Col2ER*^ mice. Result of histological showed significantly OA-like clefting and cartilage loss appeared in *Tgfbr2*^*Col2ER*^ mice. However, most notably cartilage defects were ameliorated with BSHXF treatment (Fig. 4a). Consistent with the histopathologic analysis, reduced tibial cartilage area and thickness in *Tgfbr2*^*Col2ER*^ mice were attenuated significantly by BSHXF treatment (*P *< 0.01, *P *= 0.033) (Fig. 4b, c). Furthermore, the impact of BSHXF on the articular cartilage in *Tgfbr2*^*Col2ER*^ mice was also significant difference based on OARSI scoring (*P *< 0.01) (Fig. 4d). Consistent with histological analysis, the result of COL2 IHC revealed a significantly elevated COL2 positive staining in *Tgfbr2*^*Col2ER*^ mice followed by BSHXF treatment (*P *= 0.023) (Fig. 4a, e). In addition, remarkable osteophyte formation observed in *Tgfbr2*^*Col2ER*^ mice was reduced followed by BSHXF treatment via micro-CT (Fig. 4f). Moreover, BV/TV and Tb. Th were increased, and Tb. Sp was decreased in *Tgfbr2*^*Col2ER*^ mice, was rescued by BSHXF (BV/TV, *P *= 0.035; area, *P *= 0.013; thickness, *P *= 0.010) (Fig. 4g–i).

To better understand the role of TGF-β/MMP13 signaling in the chondroprotective effect of BSHXF, we next performed IHC to evaluate TGFBR2, pSMAD2 and MMP13 protein expressional levels in *Tgfbr2*^*Col2ER*^ mice. Compared to Cre-negative mice, *Tgfbr2*^*Col2ER*^ mice revealed significantly reduced levels of TGFBR2 and pSMAD2 in articular cartilage (both, *P *< 0.01) (Fig. 4j–l). As expected, elevated MMP13 positive cells were observed in *Tgfbr2*^*Col2ER*^ mice (*P *< 0.01) (Fig. 4j, m). TGFBR2 positive cells were found slightly increased in *Tgfbr2*^*Col2ER*^ mice followed by BSHXF treatment (*P *= 0.034) (Fig. 4j, k). Interestingly, however, BSHXF treatment significantly elevated the pSMAD2 and reduced MMP13 expression levels (*P *< 0.01, *P *= 0.012) (Fig. 4j, l, m). Consistent with the IHC examination, transfection of *Tgfbr2* siRNA increased *Mmp13* expression inchondrogenic ATDC5 cells. Moreover, treatment with BSHXF significantly inhibited *Mmp13* expression (*P *< 0.01) (Fig. 4n). Additionally, we also found that BSHXF significantly reduced the level of chondrocyte apoptosis in *Tgfbr2*^*Col2ER*^ mice (*P *< 0.01) (Fig. 4o, p). All these results suggest that BSHXF had effect on inhibition of cartilage matrix degeneration as the MMP13 inhibitor through TGF-β/MMP13 signaling.

## Discussion

In the present study, we have investigated the effect of BSHXF on articular cartilage degradation in DMM-induced OA murine model. We also have observed in TGF-β/MMP13 signaling mechanism through *Tgfbr2*^*Col2ER*^ mice. Our data provided molecular evidences that BSHXF decelerates osteoarthritic cartilage degeneration performed as a MMP13 inhibitor via regulation of TGF-β/MMP13 signaling.

Reproducing characteristics of OA in animals is critical to have a better understanding of disease mechanism. DMM surgical model is one of the most common models of OA in laboratory animals, and strongly associated with clinical process [22, 23]. DMM model provided extremely good reproducibility and considered to be a gold standard in the field [24]. Histology demonstrated the cartilage damage and subchondral bone sclerosis developed 8 weeks after surgery and osteophyte formation was observed 12 weeks after surgery [18, 25, 26]. Consistent with the previous studies [18, 25, 26], DMM-induced mice in our research exhibited remarkable localized damage of articular cartilage, early osteophyte formation and increases in subchondral bone mass 12-weeks post-surgery, indicating the success of model establishment.

In OA disease, the precisely controlled balance of ECM synthesis and degeneration is disrupted, resulting in progressive erosion of articular cartilage [27]. The healthy chondrocytes play a key role for maintaining articular cartilage homeostasis partly through producing ECM components, including COL2, proteoglycan, etc. MMP13 is a major enzyme that not only degrades COL2 in cartilage, but also targets proteoglycan, osteonectin and perlecan in cartilage for degeneration [28]. Abnormal chondrocyte in osteoarthritic cartilage induced down-regulated COL2 expression in the superficial layer and up-regulated MMP13 expression [29, 30]. Notably, one of the pivotal regulatory regions of the MMP13 promoter is the RUNX2 binding site, which has been implicated in the TGF-β-inducible MMP13 expression in articular cartilage [13, 31]. Moreover, related study reported that *Tgfbr2* cKO induces development of OA via the up-regulation of RUNX2 and MMP13 in chondrocyte [13]. Consistent with the previous studies, we found reduced protein expressional level of TGFBR2 and elevated MMP13 in DMM-induced mice. These observations strongly support the notion that elevated MMP13 expression in chondrocyte through TGF-β/MMP13 signaling may play a critical role during the process of DMM-induced OA.

Such novel findings indicate that MMP13 expression in chondrocyte may represent a potential therapeutic target in OA. In preclinical testing, MMP inhibitors have inhibited the degradation of articular cartilage in animal models of OA [32, 33]. However, most clinical trials have not been continued due to concerns of dose-limiting toxicity. Although a number of hypotheses have been proposed for the toxicity, including broad-spectrum MMP inhibitors, the pharmacological basis for side effects remains poorly understood [34, 35]. Chinese medicine acts as the promising alternative medicine as a therapeutic option for OA and might be easily accepted by clinicians and patients [16]. BSHXF, a traditional Chinese Herbal formula, is composed of *Prunus persica* (Batsch.), *Carthamus tinctorius* (L.), *Rehmannia glutinosa* (Libosch.), *Eucommia ulmoides* (Oliv.), *Aconitum carmichaelii* (Debx.), *Lycium barbarum* (L.), *Cinnamomum cassia* (Presl.), *Cornus officinalis* (Sieb.), *Dioscorea opposite* (Thunb.) and *Glycyrrhiza uralensis* (Fisch.). In our study, the constituents of BSHXF were separated and defined by HPLC analysis, and six components were determined as major active material basis. Amygdalin and loganin showed highest concentrations, followed by liquiritin, pinoresinol diglucoside, hydroxysafflor yellow A and cinnamaldehyde. Our previous study had demonstrated that BSHXF may decelerate osteoarthritic cartilage degeneration [17]. Niu et al. [36] reported that combination of Amygdalin and hydroxysafflor yellow A was shown to have the potential to inhibit the degeneration of chondrocytes and better than the single use of amygdalin or hydroxysafflor yellow A, which fully embodied the TCM strengths and may partly explain the protective effect of BSHXF on articular cartilage.

In this study, significant chondroprotective effects were detected in DMM-induced mice treated with BSHXF. Our data showed that BSHXF could elevate protein expressional level of TGFBR2 and pSMAD2, and reduce MMP13 in cartilage of DMM-induced mice. To further determine the mechanism of BSHXF inhibited articular cartilage degeneration in OA disease, we investigated the efficacy of BSHXF in reversing the OA-like phenotype of *Tgfbr2*^*Col2ER*^ mice. As reported in previous study, TGF-β signaling regulates the expression of genes that are important in the maintenance of ECM [13]. Inactivation of TGF-β signaling promotes *Mmp13* gene transcription in a RUNX2-dependent manner [13]. In addition to mutation of *Tgfbr2* gene, potential causes of TGF-β signaling inactivation in articular cartilage include mutation of other genes responsible for mediating TGF-β signaling, such as *Smad3* and *p38* [31]. In our study, the loss of articular cartilage observed in *Tgfbr2*^*Col2ER*^ mice was rescued by BSHXF treatment through its suppressive effect on the expression of MMP13. However, we found that BSHXF treatment slightly elevated the TGFBR2 expression level although *Tgfbr2* gene was specifically knocked out in articular cartilage. One possible reason for this finding is that about 80% TGFBR2 proteins were blocked in articular cartilage of *Tgfbr2*^*Col2ER*^ mice. And the Cre recombination efficiency mediated by *Col2*-*CreER* is close to the previous study [13]. In other words, TGFBR2 activity in the other 20% of chondrocytes remains normal in *Tgfbr2*^*Col2ER*^ mice. Thus, the rescue of BSHXF treatment on chondroprotective effects was incomplete. So we reasoned that it may be compensated for by regulation of the downstream gene expression in TGF-β signaling. Interestingly, *Tgfbr2*^*Col2ER*^ mice supplemented with BSHXF had significant elevated protein expression level of pSMAD2 in chondrocytes compared to control *Tgfbr2*^*Col2ER*^ mice. And this finding was consistent with the previous study [31]. Overall, these discoveries suggested that preservation of cartilage architecture in BSHXF treatment groups could be due to reduced MMP13 production by chondrocytes via activating TGF-β/MMP13 signaling.

## Conclusions

In conclusion, our study revealed that BSHXF attenuates osteoarthritic articular cartilage degradation as a natural MMP13 inhibitor through TGF-β/MMP13 signaling in articular cartilage. These findings serve to underscore the potential value of TCM in the treatment of chronic OA disease and encourage further research into this therapeutic modality.

## Abbreviations

BSHXF: Bushenhuoxue formula
OA: osteoarthritis
MMTL: medial meniscotibial ligament
DMM: destabilization of the medial meniscus
EMC: extracellular matrix content
NSAIDs: non-steroidal anti-inflammatory drugs
COX-II: cyclooxygenase-2
COL2: collagen II
MMP13: metalloproteinase 13
TGF-β: transforming growth factor beta
TGFBR2: transforming growth factor beta receptor type II
TCM: traditional Chinese medicine
HPLC: high-performance liquid chromatography
cKO: conditional knockout
OARSI: Osteoarthritis Research Society International
WT: wild type
TUNEL: terminal deoxynucleotidyl transferase dUTP nick end labeling
IHC: immunohistochemistry
real-time PCR: real-time polymerase chain reaction
siRNA: small interfering RNA
SD: Sprague–Dawley
LSD: least significant difference

## References

[1] Bomer N, den Hollander W, Ramos YF, Meulenbelt I (2015). Translating genomics into mechanisms of disease: osteoarthritis. Best Pract Res Clin Rheumatol.

[2] Cross M, Smith E, Hoy D, Nolte S, Ackerman I, Fransen M (2014). The global burden of hip and knee osteoarthritis: estimates from the global burden of disease 2010 study. Ann Rheum Dis.

[3] Vaishya R, Pariyo GB, Agarwal AK, Vijay V (2016). Non-operative management of osteoarthritis of the knee joint. J Clin Orthop Trauma.

[4] Veronesi F, Della BE, Cepollaro S, Brogini S, Martini L, Fini M (2016). Novel therapeutic targets in osteoarthritis: narrative review on knock-out genes involved in disease development in mouse animal models. Cytotherapy.

[5] Lietman SA, Miyamoto S, Brown PR, Inoue N, Reddi AH (2002). The temporal sequence of spontaneous repair of osteochondral defects in the knees of rabbits is dependent on the geometry of the defect. J Bone Joint Surg Br.

[6] Correa D, Lietman SA (2017). Articular cartilage repair: current needs, methods and research directions. Semin Cell Dev Biol.

[7] Roach HI, Yamada N, Cheung KS, Tilley S, Clarke NM, Oreffo RO (2005). Association between the abnormal expression of matrix-degrading enzymes by human osteoarthritic chondrocytes and demethylation of specific CpG sites in the promoter regions. Arthritis Rheum.

[8] Neuhold LA, Killar L, Zhao W, Sung ML, Warner L, Kulik J (2001). Postnatal expression in hyaline cartilage of constitutively active human collagenase-3 (MMP-13) induces osteoarthritis in mice. J Clin Investig.

[9] Blaney DEN, van der Kraan PM, van den Berg WB (2007). TGF-beta and osteoarthritis. Osteoarthr Cartil.

[10] Quintana L, zur Nieden NI, Semino CE (2009). Morphogenetic and regulatory mechanisms during developmental chondrogenesis: new paradigms for cartilage tissue engineering. Tissue Eng Part B Rev.

[11] Cuellar A, Reddi AH (2015). Stimulation of superficial zone protein/lubricin/PRG4 by transforming growth factor-beta in superficial zone articular chondrocytes and modulation by glycosaminoglycans. Tissue Eng Part A.

[12] Yang X, Chen L, Xu X, Li C, Huang C, Deng CX (2001). TGF-beta/Smad3 signals repress chondrocyte hypertrophic differentiation and are required for maintaining articular cartilage. J Cell Biol.

[13] Shen J, Li J, Wang B, Jin H, Wang M, Zhang Y (2013). Deletion of the transforming growth factor beta receptor type II gene in articular chondrocytes leads to a progressive osteoarthritis-like phenotype in mice. Arthritis Rheum.

[14] Baragi VM, Becher G, Bendele AM, Biesinger R, Bluhm H, Boer J (2009). A new class of potent matrix metalloproteinase 13 inhibitors for potential treatment of osteoarthritis: evidence of histologic and clinical efficacy without musculoskeletal toxicity in rat models. Arthritis Rheum.

[15] Li NG, Tang YP, Duan JA, Shi ZH (2014). Matrix metalloproteinase inhibitors: a patent review (2011–2013). Expert Opin Ther Pat.

[16] Li L, Liu H, Shi W, Liu H, Yang J, Xu D (2017). Insights into the action mechanisms of traditional Chinese medicine in osteoarthritis. Evid Based Complement Altern Med.

[17] Ji WF, Shi WF, Chen L, Ma ZC, Yuan XF, Xu J (2012). Experimental study on invigorating kidney and activating blood on preventing and curing SD rats with knee osteoarthritis. Zhongguo Gu Shang.

[18] Glasson SS, Askew R, Sheppard B, Carito B, Blanchet T, Ma HL (2005). Deletion of active ADAMTS5 prevents cartilage degradation in a murine model of osteoarthritis. Nature.

[19] Xu SY, Bian RL, Chen X, Liu GQ, Ling SS, Wei W, Sun RY, Ma YM, Hong ZY (2002). Dose problems in pharmacological experiments. Experimental methodology of pharmacology.

[20] Glasson SS, Chambers MG, Van Den Berg WB, Little CB (2010). The OARSI histopathology initiative—recommendations for histological assessments of osteoarthritis in the mouse. Osteoarthr Cartil.

[21] Hildebrand T, Laib A, Muller R, Dequeker J, Ruegsegger P (1999). Direct three-dimensional morphometric analysis of human cancellous bone: microstructural data from spine, femur, iliac crest, and calcaneus. J Bone Miner Res.

[22] Glasson SS, Blanchet TJ, Morris EA (2007). The surgical destabilization of the medial meniscus (DMM) model of osteoarthritis in the 129/SvEv mouse. Osteoarthr Cartil.

[23] Iijima H, Aoyama T, Ito A, Tajino J, Nagai M, Zhang X (2014). Destabilization of the medial meniscus leads to subchondral bone defects and site-specific cartilage degeneration in an experimental rat model. Osteoarthr Cartil.

[24] Culley KL, Dragomir CL, Chang J, Wondimu EB, Coico J, Plumb DA (2015). Mouse models of osteoarthritis: surgical model of posttraumatic osteoarthritis induced by destabilization of the medial meniscus. Methods Mol Biol.

[25] Ma HL, Blanchet TJ, Peluso D, Hopkins B, Morris EA, Glasson SS (2007). Osteoarthritis severity is sex dependent in a surgical mouse model. Osteoarthr Cartil.

[26] Welch ID, Cowan MF, Beier F, Underhill TM (2009). The retinoic acid binding protein CRABP2 is increased in murine models of degenerative joint disease. Arthritis Res Ther.

[27] Bonet ML, Granados N, Palou A (2011). Molecular players at the intersection of obesity and osteoarthritis. Curr Drug Targets.

[28] Shiomi T, Lemaitre V, D’Armiento J, Okada Y (2010). Matrix metalloproteinases, a disintegrin and metalloproteinases, and a disintegrin and metalloproteinases with thrombospondin motifs in non-neoplastic diseases. Pathol Int.

[29] Aigner T, Vornehm SI, Zeiler G, Dudhia J, Von der Mark K, Bayliss MT (1997). Suppression of cartilage matrix gene expression in upper zone chondrocytes of osteoarthritic cartilage. Arthritis Rheum.

[30] Shlopov BV, Lie WR, Mainardi CL, Cole AA, Chubinskaya S, Hasty KA (1997). Osteoarthritic lesions: involvement of three different collagenases. Arthritis Rheum.

[31] Chen CG, Thuillier D, Chin EN, Alliston T (2012). Chondrocyte-intrinsic Smad3 represses Runx2-inducible matrix metalloproteinase 13 expression to maintain articular cartilage and prevent osteoarthritis. Arthritis Rheum.

[32] Sabatini M, Lesur C, Thomas M, Chomel A, Anract P, de Nanteuil G (2005). Effect of inhibition of matrix metalloproteinases on cartilage loss in vitro and in a guinea pig model of osteoarthritis. Arthritis Rheum.

[33] Wang M, Sampson ER, Jin H, Li J, Ke QH, Im HJ (2013). MMP13 is a critical target gene during the progression of osteoarthritis. Arthritis Res Ther.

[34] Hoekstra R, Eskens FA, Verweij J (2001). Matrix metalloproteinase inhibitors: current developments and future perspectives. Oncologist.

[35] Peterson JT (2006). The importance of estimating the therapeutic index in the development of matrix metalloproteinase inhibitors. Cardiovasc Res.

[36] Niu K, Zhao YJ, Zhang L, Li CG, Wang YJ, Zheng WC (2014). The synergistic effect of amygdalin and HSYA on the IL-1beta induced endplate chondrocytes of rat intervertebral discs. Yao Xue Xue Bao.

